# Next Generation Vaccines for Infectious Diseases

**DOI:** 10.1155/2019/5890962

**Published:** 2019-04-30

**Authors:** Giuseppe A. Sautto, Greg A. Kirchenbaum, Roberta A. Diotti, Elena Criscuolo, Francesca Ferrara

**Affiliations:** ^1^Center for Vaccines and Immunology, University of Georgia, Athens, GA, USA; ^2^Cellular Technology Ltd., Shaker Heights, OH, USA; ^3^Microbiology and Virology Unit, “Vita-Salute San Raffaele” University, Milan, Italy; ^4^Pomona Ricerca S.r.l., Turin, Italy; ^5^Vector Development and Production Laboratory, St. Jude Children's Research Hospital, Memphis, TN, USA

Vaccines represent the most effective prophylactic strategy in our arsenal for controlling the spread of infectious diseases and have increased human life expectancy. As an example, the eradication of smallpox and the massive reduction of other infectious diseases such as polio, measles, and diphtheria represent major vaccination victories of the last century. Despite the important successes achieved through vaccination, ongoing efforts continue towards development of new and more protective vaccines. Namely, a number of challenges still remain in the context of vaccination. Specifically, many commercially available vaccines fail to elicit long-lasting immune responses and insufficiently trigger cell-mediated and mucosal immunity. Moreover, many vaccines remain dependent on a cold chain for maintenance of antigenicity and potency. Finally, vaccine compliance remains an issue in our society.

Most often, vaccines are composed of attenuated or inactivated pathogens and are capable of eliciting a protective immune response while avoiding the complications associated with an infection. In recent years, thanks to advances in biotechnology and improvements in the production of recombinant proteins, other vaccine strategies have been developed and approved for human use. Many other vaccines produced using recombinant technologies are currently under development and evaluation in clinical trials. As an example, the HBV, HPV, and serogroup B meningococcal vaccines currently in use are composed of subunits that focus the immune response towards specific components of the pathogen.

Moreover, to improve vaccine elicitation of a cell-mediated and mucosal immunity, new DNA-based, viral vector-based, and other novel vaccine delivery platforms, along with usage of adjuvants and chemotactic agents, have been developed. In this regard, N. Cotugno et al. describe the use of OMIC techniques to identify specific genomic profiles defining which vaccinal system might be used in nonresponder individuals. E. Criscuolo et al. review the most recent advances in edible vaccine systems and intradermal vaccine delivery. These new vaccination strategies represent significant advancements for vaccine stability considering the fact that they do not require a cold chain. E. N. Gary and M. A. Kutzler discuss the use of chemokines as adjuvant for inducing mucosal immunity. O. Koutsoni et al. describe how a new adjuvant, the Leishmania eukaryotic Initiation Factor, can be considered both as a natural adjuvant and as an antigen, highlighting its immunomodulatory properties *in vitro* and *in vivo*.

Additional challenges towards reducing the transmission of infectious diseases remain. As such, controlling the spread of these pathogenic microorganisms in animal reservoirs is another important step towards complete eradication. In this regard, vaccination of domestic poultry and livestock is an important tool for maintaining animal health but is also essential for reducing spread of zoonotic pathogens. Nipah virus infection, severe acute respiratory syndrome, and Highly Pathogenic Avian Influenza (HPAI) are a few examples of infectious diseases originating with transmission from wild animal reservoirs to livestock and then to humans. The development of highly effective vaccines for livestock animals is therefore an essential block for transmission of zoonotic pathogens to humans. In an experimental challenge and transmission model, V. Palya et al. show that vector-based vaccines can efficiently protect commercial broilers and layer pullets from HPAI virus infection.

Especially in underdeveloped countries, affordability of new generation vaccines will be critically important for reducing disease transmission in both human and livestock species. As a necessary step in this direction, Y. Burakova et al. describe the development of cost-effective veterinary vaccine formulations.

In this open special issue, recent advances in the design, formulation, and delivery of vaccines against infectious diseases are covered by three review articles and nine research papers. As guest editors of this open special issue, we hope that readers find its content interesting and that these works inspire continuous efforts towards development and implementation of improved vaccines.

